# Manual of Operative Surgery

**Published:** 1887-05

**Authors:** 


Manual of Operative Surgery. *By* Joseph D. Bryant, M.D., *Professor of Anatomy and Clinical Surgery, etc., Bellevue Hospital Medical College, etc., etc., pp. xxvi, and 530, quarto, with 791 illustrations. New York:* D. Appleton & Co. *Chicago:* A. C. McClurg & Co. *1887.*

The very profuse illustrations in this book are a great aid to a text which is clear and concise. The intention of the author was to assist the student in the acquisition of established facts in operative surgery, rather han write an exhaustive treatise. Operations peculiar to the female sex, and those of the eye and of the ear, are omitted, as demanding more extensive consideration than the scope of this treatise allows.

The remarks in connection with the choice and employment of anæsthetics are in accordance with the ideas upon that subject now prevalent n the United States.

The author's style is pleasant, and the description of the various operations is very clear. The book is evidently written by a successful teacher, and it can hardly fail to promptly become the leading text-book upon the subject of which it treats. The letter-press is excellent, and deserves commendation.

